# Impact of yeast and lactic acid bacteria on mastitis and milk microbiota composition of dairy cows

**DOI:** 10.1186/s13568-020-0953-8

**Published:** 2020-01-29

**Authors:** Jing Gao, Yu-Chen Liu, Yu Wang, Han Li, Xiang-Ming Wang, Yan Wu, Ding-Ran Zhang, Si Gao, Zhi-li Qi

**Affiliations:** Department of Animal Nutrition and Feed Science, College of Animal Science and Technology, Huazhong Agricultural University, Wuhan, China

**Keywords:** Lactic acid bacteria, Yeast, Mastitis, Milk microbiota composition, Dairy cows

## Abstract

This experiment was conducted to evaluate the impact of yeast and lactic acid bacteria (LAB) on mastitis and milk microbiota composition of dairy cows. Thirty lactating Holstein cows with similar parity, days in milk were randomly assigned to five treatments, including: (1) Health cows with milk SCC < 500,000 cells/mL, no clinical signs of mastitis were found, fed basal total mixed ration (TMR) without supplementation (H); (2) Mastitis cows with milk SCC > 500,000 cells/mL, fed basal TMR without supplementation (M); (3) Mastitis cows fed basal TMR supplemented with 8 g day^−1^ yeast (M + Y); (4) Mastitis cows fed basal TMR supplemented with 8 g day^−1^ LAB (M + L); (5) Mastitis cows (milk SCC > 500,000 cells/mL) fed basal TMR supplemented with 4 g day^−1^ yeast and 4 g day^−1^ LAB (M + Y + L). Blood and milk sample were collected at day 0, day 20 and day 40. The results showed efficacy of probiotic: On day 20 and day 40, milk SCC in H, M + Y, M + L, M + Y + L was significantly lower than that of M (*P *< 0.05). Milk concentration of TNF-α, IL-6 and IL-1β in M + Y + L were significantly reduced compared with that of M on day 40 (*P *< 0.05). Milk Myeloperoxidase (MPO) and *N*-Acetyl-β-d-Glucosaminidase (NAG) activity of M + Y, M + L, M + L + Y were lower than that of M on day 40 (*P *< 0.05). At genus level, *Staphylococcus, Chryseobacterium* and *Lactococcus* were dominant. Supplementation of LAB decreased abundance of *Enterococcus* and *Streptococcus,* identified as mastitis-causing pathogen. The results suggested the potential of LAB to prevent mastitis by relieving mammary gland inflammation and regulating milk microorganisms.

## Introduction

Mastitis, characterized by high amounts of bacteria and mammary inflammation, is one of the most frequent disease occurred on dairy cows and has been well-recognized detrimental effects on milk quality, animal well-being and public health. A variety of bacteria pathogens were identified to be involved in the development of mastitis. *Streptococcus agalactiae* and *Staphylococcus* was considered as the mainly contagious pathogens (Ruegg 2017). Murphy (1947) demonstrated the general process of mastitis, which bacteria being established in the mammary gland as initial and followed by inflammation.

Intramammary administration of antibiotics is the widely used conventional therapy for clinical mastitis during lactating and also for prevention of new infections during dry-off. Despite the effectiveness of controlling mastitis, the development of antimicrobial resistance caused by common use of antibiotics raises society concern. In order to reduce antibiotic residues in dairy products and coincide with global requirement to limit their use in dairy cattle, the use of probiotic agents is a novel approach to cure mastitis. Probiotics are live microorganisms, which confer a health benefit to the host when administered in adequate amounts (FAO and WHO 2008). Researches have demonstrated the ability of probiotics to protect against microbial pathogens and enhance the immune functions (Cross 2002). The use of probiotics bacteria mainly focuses on gastrointestinal, vaginal tract and mammary gland (Frola et al. 2012; Deng et al. 2015; Walsh et al. 2010). The mechanism that probiotics exert beneficial effects on host health is closely associated with the capability to produce antagonistic substances, adhesion to host tissues and colonization to different sites of the host surfaces (Espeche et al. 2009). However, the dietary supplementation of probiotic on immune system and mastitis is unknown, which is more practical in dairy industrial compared with appliance to gastrointestinal tract and mammary gland. Yeast supplementation on ruminants has been shown to regulate rumen pH, microbial composition and fiber fermentation (Bach et al. 2018; Terre et al. 2015). Recent studies suggest the supplementation of yeast may also influence immune function. Zanello et al. (2011) reported that yeast inhibit the *Escherichia coli*—induced expression of pro-inflammation transcripts and protein, including IL-6, IL-8. Furthermore, yeast also moderate recruitment and activation of immune cells in differentiated porcine intestinal epithelial cells. Yuan et al. (2015) analyzed the effects of dietary yeast supplementation on uterine inflammatory and they found that although no treatment effect was detected for incidence of subclinical endometritis, supplementary yeast decreased uterine IL-6 mRNA abundance and improved neutrophil myeloperoxidase and neutrophil elastase expression on transition cow, as well as fecal IgA concentration, which indicating relieved uterine inflammation and strengthened immune function. To our knowledge, no study has evaluated the effects of dietary supplementation of live yeast on mastitis and milk microorganism based on the high-throughput sequencing, a new method that provide a more complete relative qualification of micro community composition. Lactic acid bacteria (LAB) is also shown as potential probiotics for therapeutic use against endometrial inflammation and mastitis and prevention for diarrhea in calf, which is capable for the ability to strengthen systemic immune function (Genis et al. 2017; Bouchard et al. 2015; Maldonado et al. 2017). Intramammary LAB incubation increased the amounts of IgG isotypes in blood and milk, as well as lymphocyte proliferation and negative the major bovine mastitis pathogens on dry-off cows, which suggests restored balance in microbiota of the mammary gland and improved systemic immune function (Pellegrino et al. 2017). However, the effects of dietary supplementation of LAB and yeast on mastitis and milk microorganisms of lactating cow is unknown. Therefore, the objective of this study is to evaluate the effect of dietary supplementation of LAB and yeast on mastitis and milk microorganism based on the high-throughput sequencing.

## Materials and methods

### Animals and experimental design

Thirty lactating Holstein cows with similar parity (1.5 ± 0.3) and days in milk (145 ± 2) were selected and assigned to five treatments, 6 cows each treatment, including: (1) Health cows with milk SCC < 500,000 cells/mL, no clinical signs of mastitis were found in four mammary quarters and no incidence of clinical mastitis during the last 90 days of the previous lactation, fed basal total mixed ration (TMR) without supplementation (H); (2) Mastitis cows with milk SCC > 500,000 cells/mL, fed basal TMR without supplementation (M); (3) Mastitis cows (milk SCC > 500,000 cells/mL) fed basal TMR supplemented with 8 g day^−1^ yeast (M + Y, company product); (4) Mastitis cows (milk SCC > 500,000 cells/mL) fed basal TMR supplemented with 8 g day^−1^ lactic acid bacteria (M + L, company products); (5) Mastitis cows (milk SCC > 500,000 cells/mL) fed basal TMR supplemented with 4 g day^−1^ yeast and 4 g day^−1^ lactic acid bacteria (M + Y + L). The yeast was *Saccharomyces cerevisiae*, with moisture contents < 6.0% and counts around 2 × 10^10^ CFU/g; LAB consisted of *Lactococcal* and maltodextrin, with moisture contents < 8.0% and counts around 2 × 10^9^ CFU/g. Cows were housed in a free-stall barn and managed in the same manner throughout the experiment. During the morning feeding, yeast chromium and dihydropyridine were mixed with 2 kg TMR and then fed to each cow based on treatments. After the TMR mixed with supplements was consumed, the remaindering portion of TMR was delivered. Cows remained on the treatments for 40 days. Cows had ad libitum access to water and were fed at 0700, 1700 h each day. Cows were milked at 0400, 1600 h.

### Sample collection and laboratory analysis

Blood sample were collected from coccygeal vessels into vacutainers without anticoagulant on day 0 and day 40 before feeding (0600 h). Blood was allowed to clot at room temperature for 20 min before centrifugation at 3000*g* for 30 min to collect serum and stored at − 20 °C until analysis. Concentration of serum immunoglobulin (IgA, IgG, IgM) were measured by commercially available ELISA kits (Jiancheng Biochemical Reagent Company, Nanjing, China) following the manufacturer’s instructions. Milk samples were collected from two consecutive milking and mixed equally at day 0, day 20 and day 40. Part of milk samples were analyzed for milk percentage of fat, protein, fat to protein ration, lactose, urea nitrogen, solid not fat and SCC. The other whole milk was centrifuged 8000*g* for 10 min and the supernatant concentration of TNF-α, IL-6, IL-1β and activity of lactic dehydrogenase (LDH), Myeloperoxidase (MPO), *N*-Acetyl-β-d-Glucosaminidase (NAG), alkaline phosphatase (ALP) were measured by Elisa as manufacturer’s instructions (Jiancheng Biochemical Reagent Company, Nanjing, China).

### DNA extraction, PCR amplification and high-throughput sequencing

On day 40, 20 milk samples were collected (4 cows each treatment) and centrifuged. The pellet was subjected to genomic DNA extraction using GenElute™ Bacterial Genomic DNA Kit (Sigma-Aldrich, Sigma Chemical Co., St. Louis, MO. USA), according to the manufacturer’s protocols. Final DNA concentration and purification were determined using a NanoDrop 2000 UV–vis spectrophotometer (Thermo Scientific, Wilmington, USA), and DNA quality was checked using 1% agarose gel electrophoresis. The bacterial primer set of forward primer 338F (5′-ACTCCTACGGGAGGCAGCAG-3′) and reverse primer 806R (5′-GGACTACHVGGGTWTCTAAT-3′) were used to amplify DNA fragments of the V1–V2 region of bacterial16S rRNA genes. TransStart Fastpfu DNA Polymerase and thermocycler PCR systems (GeneAmp 9700, ABI, USA) were available for PCR. PCR was performed triplicate in a 20 μL triplicate mixture: 4 μL of FastPfu Buffer, 2 μL of 2.5 mM dNTPs, 0.8 μL of each primer (5 μM), 0.4 μL of FastPfu Polymerase, 10 ng of template DNA, add dH_2_O to 20 μL. The PCR reaction were conducted using following program: an initial denaturation step at 95 °C for 3 min, followed by 27 cycles of denaturation at 95 °C for 30 s, annulation at 55 °C for 30 s, elongation at 72 °C for 45 s with a final extension step at 72 °C for 10 min which was halted at 10 °C. The resulting PCR products were extracted from a 2% agarose gel and further purified using an AxyPrep DNA Gel Extraction Kit (Axygen Biosciences, Union City, CA, USA) and were quantified using a QuantiFluor-ST Real-time PCR System (Promega, USA) according to the manufacturer’s protocol, before sequencing. 16S rRNA gene sequencing was performed on an Illumina Miseq 300 Platform (Illumina, San Diego, USA) according to standard protocols by Majorbio Bio-pharm Technology Co., Ltd., Shanghai, China). The raw reads were deposited into Zenodo database (http://doi.org/10.5281/zenodo.3384327).

### Data analysis

Serum concentration of IgA, IgM, IgG and milk composition, concentration of TNF-α, IL-1β, IL-6 and activity of LDH, MPO, NAG, ALP were subjected were subjected to analysis of variance (ANOVA) using SPSS statistical software (SPSS 17.0). Statistical differences among means (*P* < 0.05) were identified using Duncan’s multiple range test. Significance was declared at *P* < 0.05. Means and SEM are reported. Microbial community and group difference analyses were performed using the free online platform, Majorbio I-Sanger Cloud Platform (www.i-sanger.com).

## Results

### Milk composition

On day 0, cows in M, M + Y, M + L, M + Y + L had significantly higher milk SCC compared with that of H (*P *< 0.05, Table 1). On day 20 and day 40, milk SCC in H, M + Y, M + L, M + Y + L was significantly lower than that of M on day 20 and day 40 (*P *< 0.05). Milk percentage of fat in M was significantly reduced compared with that of H and M + Y + L on day 20 and day 40 (*P *< 0.05). Cows in M + Y + L had significantly higher percentage of milk protein than cows in other treatments on day 40 (*P *< 0.05). Milk fat to protein ratio and percentage of lactose in M + Y, M + L, M + Y + L was significantly increased compared with that of M on day 40 (*P *< 0.05). Cows in H, M + Y + L had higher milk percentage of urea nitrogen than cows in M on day 20 and day 40 (*P *< 0.05). There were no treatment effects on milk percentage of solid not fat (*P *> 0.05).

**Table 1:** The effect of treatments on milk composition

Items	Time	Treatments					SEM	*P*-value
		H	M	M + Y	M + L	M + Y + L		
SCC (10^4^/mL)	Day0	42.00^b^	229.42^a^	199.33^a^	217.83^a^	247.16^a^	21.75	0.05
	Day20	65.25^b^	352.14^a^	136.50^b^	174.66^b^	172.50^b^	29.71	0.02
	Day40	56.25^B^	294.42^A^	112.83^B^	129.67^B^	121.33^B^	24.65	0.01
Milk fat (%)	Day0	3.22	2.68	2.20	2.47	2.83	0.12	0.16
	Day20	3.37^A^	2.00^B^	2.76^AB^	2.69^AB^	3.41^A^	0.15	0.01
	Day40	3.23^B^	2.65^B^	3.30^B^	3.40^B^	4.23^A^	0.15	0.01
Milk protein (%)	Day0	3.35	3.52	3.23	3.29	3.58	0.06	0.34
	Day20	3.56	3.65	3.37	3.42	3.70	0.06	0.33
	Day40	3.62^b^	3.56^b^	3.46^b^	3.61^b^	4.02^a^	0.07	0.05
Fat to protein ratio	Day0	0.96	0.75	0.68	0.74	0.78	0.03	0.17
	Day20	0.94^A^	0.54^B^	0.82^A^	0.78^A^	0.90^A^	0.04	0.00
	Day40	0.89^ab^	0.74^b^	0.96^a^	0.94^a^	1.04^a^	0.04	0.05
Lactose (%)	Day0	4.87	5.09	4.86	5.08	4.93	0.05	0.49
	Day20	5.21	4.94	5.01	5.19	5.03	0.04	0.18
	Day40	5.04^B^	4.80^B^	5.12^A^	5.28^A^	5.35^A^	0.05	0.00
Urea nitrogen (%)	Day0	12.12	12.60	11.43	11.55	11.51	0.30	0.68
	Day20	12.65^a^	10.67^b^	11.68^b^	11.65^b^	13.41^a^	0.30	0.02
	Day40	13.82^a^	11.70^b^	13.50^a^	12.71^ab^	13.71^a^	0.28	0.07
Solids not fat (%)	Day0	9.06	9.43	8.87	9.17	9.18	0.08	0.23
	Day20	9.59	9.39	9.23	9.34	9.25	0.07	0.59
	Day40	9.42	9.54	9.40	9.41	9.61	0.08	0.89

In dairy cows, SCC and milk of inferior are important predictors for mastitis

^abc^Means in the same row with significant difference (*P* < 0.05)

^ABC^Means in the same row with extremely significant difference (*P* < 0.01)

### Serum immunoglobulin

As shown in Table 2, Cows in M + Y had lower serum concentration of IgA and IgM compared with that of M on day 40 (*P *< 0.05). Serum concentration of IgG in M + Y, M + L and M + Y + L was significantly deceased compared with that of M on day 40 (*P *< 0.01).

**Table 2:** The effect of treatments on serum concentration of immunoglobulin and milk concentration of inflammation cytokine and enzyme activity

Items	Time	Treatments					SEM	*P*-value
		H	M	M + Y	M + L	M + Y + L		
IgA (ng/mL)	Day0	8.52^C^	8.92^BC^	8.93^BC^	10.31^AB^	11.23^A^	0.24	0.00
	Day40	9.07^ab^	9.77^b^	8.11^a^	9.20^ab^	9.66^b^	0.19	0.03
IgM (ng/mL)	Day0	13.20^C^	16.25^B^	17.48^AB^	19.02^A^	18.52^AB^	0.46	0.00
	Day40	14.04^a^	17.34^b^	15.04^a^	15.94^ab^	15.60^ab^	0.32	0.01
IgG (ng/mL)	Day0	15.64^b^	19.48^a^	19.82^a^	18.39^a^	18.98^a^	0.47	0.03
	Day40	18.27^AB^	20.42^B^	16.67^A^	15.86^A^	16.66^A^	0.43	0.00
TNF-α (pg/mL)	Day0	85.26	95.85	105.39	108.47	96.08	3.17	0.16
	Day40	91.30^AB^	117.34^A^	82.87^B^	87.68^B^	80.46^B^	3.87	0.01
IL-6 (pg/mL)	Day0	80.80^B^	97.88^AB^	104.45^AB^	123.08^A^	105.37^AB^	3.81	0.00
	Day40	83.87^AB^	107.90^A^	86.30^AB^	91.69^AB^	59.67^B^	4.33	0.00
IL-1β (pg/mL)	Day0	157.63^b^	180.19^ab^	207.53^a^	179.76^ab^	185.76^ab^	4.89	0.02
	Day40	161.612^ab^	188.67^a^	162.88^ab^	160.34^ab^	143.54^b^	4.85	0.04
LDH (U/L)	Day0	3533.73^c^	4129.25^bc^	6507.89^a^	6051.62^ab^	5130.87^abc^	342.71	0.02
	Day40	4529.75	5661.56	4484.27	3954.33	4029.85	231.52	0.10
ALP (U/L)	Day0	161.58	206.63	225.91	227.91	225.55	1.75	0.44
	Day40	212.77	282.17	187.85	170.22	150.59	2.59	0.16
MPO (U/L)	Day0	29.01^b^	38.35^ab^	38.89^ab^	44.17^a^	33.94^ab^	1.66	0.04
	Day40	36.38^B^	47.06^A^	34.11^B^	36.67^B^	29.50^B^	1.65	0.00
NAG (U/L)	Day0	26.21^B^	111.13^A^	63.48^AB^	115.84^A^	59.71^AB^	8.33	0.00
	Day40	63.72^BC^	130.80^A^	40.00^CD^	84.27^B^	27.59^D^	8.49	0.00

H: healthy cows without mastitis fed basal diet; M: cows with mastitis fed basal diet; M + Y: cows with mastitis fed basal diet supplemented with 8 g day^−1^ yeast; M + L: cows with mastitis fed basal diet supplemented with 8 g day^−1^ lactic acid bacteria; M + Y + L: cows with mastitis fed basal diet supplemented with 4 g day^−1^ yeast and 4 g day^−1^ lactic acid bacteria

^abc^Means in the same row with significant difference (*P* < 0.05)

^ABC^Means in the same row with extremely significant difference (*P* < 0.01)

### Milk inflammation cytokine and enzyme activity

Milk concentration of TNF-α in M + Y, M + L, M + Y + L significantly reduced compared with that of M on day 40 (*P *< 0.01, Table 2). Cows in M + Y + L had significantly lower milk concentration of IL-6 and IL-1β than cows in M on day 40 (*P *< 0.05). While there was no difference among other treatments (*P *> 0.05). Milk LDH activity in M + Y and M + L was significantly higher than that of H on day 0 (*P *< 0.05), while there was no significant difference between groups on day 40 (*P *> 0.05). Treatments didn’t affect the activity of ALP on day 0 and day 40 (*P *> 0.05). Milk MPO and NAG activity of M + Y, M + L, M + L + Y declined compared to M on day 40 (*P *< 0.05).

### General DNA sequencing observation

**Fig. 1:**
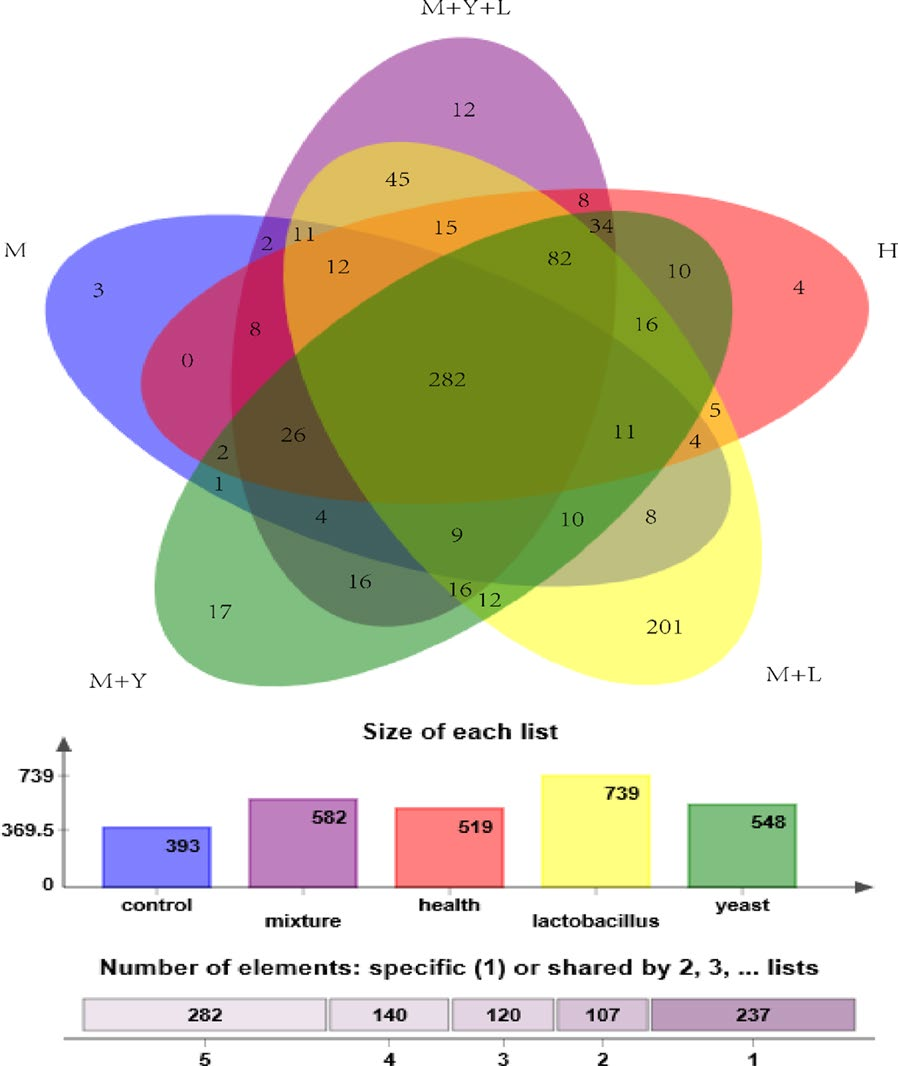
Venn diagram of OUTs from different treatments

In total, 909,264 bacterial raw 16 rRNA sequences were gained for the 20 milk samples from 5 treatments and at least 37,799 sequences were obtained per sample. Sequences with higher than 97% similarity were classified as OUT. The rarefaction curve generated reached stable level, suggesting that the number of OTUs did not increase with the expending volume of data (Additional file 1: Fig. S1). In current study, a total of 886 OUT were detected. The number of OUT shared by 5 treatments were 282, accounting for 31.82% of total OUT. The number of unique OUT for H, M, M + Y, M + L, M + Y + L were 4, 3, 17, 201 and 12 respectively (Fig. 1).

### Alpha and beta diversities of milk microbiota

**Fig. 2:**
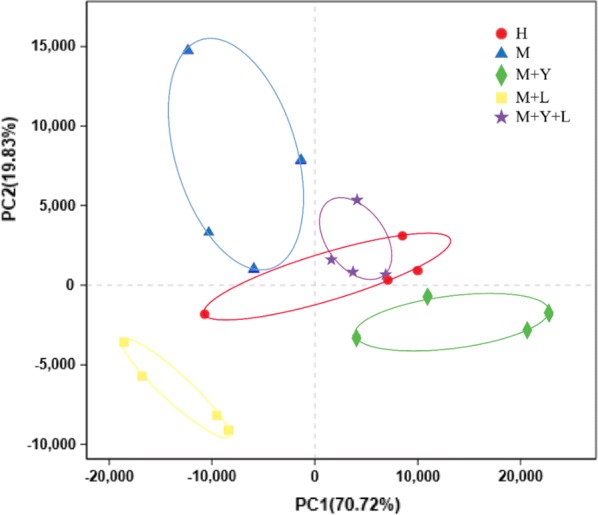
PCA analysis of milk samples microbiota of different treatments on genus level

**Fig. 3:**
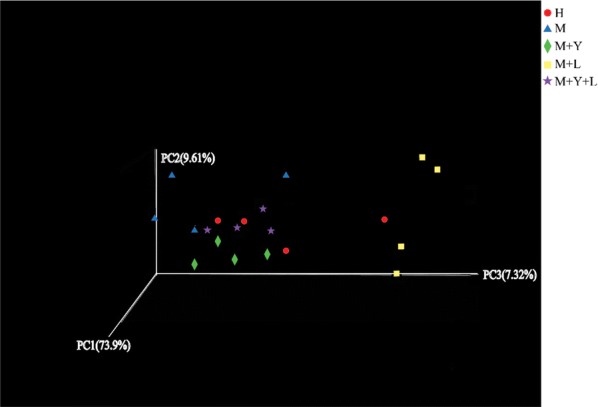
Scatterplots from principal coordinates analysis (PCoA), based on weighted UniFrac distance in bacterial communities

Alpha diversity can be used to measure the microbiota diversity and richness. As was shown in Table 3, there was no difference on Shannon and Simpson index among groups (*P* > 0.05). The Ace and Chao index of M were significantly lower than that of other treatments (*P* < 0.05). Beta diversity can determine the microbiota diversity between different samples. PCA was conducted using genus-level taxonomic profiles to compare the composition diversity (Fig. 2). Samples from groups M, M + Y, M + L, M + Y + L were able to separate based on their clustering of microbiota, suggesting a shift in the milk microbial communities with the oral supplementation of yeast and LAB. However, there was no clear separation of milk samples from groups H and M + Y + L because of the overlap. Principal coordinate analysis (PCoA) based on weighted UniFrac distances was performed for each group and the emperor PCoA scatterplot obtained are depicted in Fig. 3. The R^2^ was measured to test for the percentage of variation among samples in each group using Adonis. The mean distances among the five treatments were calculated and were shown to be significantly different in the weighted analysis (R^2^ = 0.7114, *P *= 0.005), indicating a qualitatively different composition of the milk microbiota caused by the treatments.

**Table 3:** Diversity indices and richness estimators of the 16rRNA gene libraries in milk microbiota of dairy cows in different treatments

Items	Treatments					SEM	*P*-value
	H	M	M + Y	M + L	M + Y + L		
Sequence	49,642	47,709	46,315	40,192	44,501	2216	0.13
Shannon	2.65	2.41	2.18	2.53	2.73	0.40	0.70
Simpson	0.21	0.19	0.28	0.20	0.16	0.07	0.56
Ace	451.51^a^	305.84^b^	491.65^a^	509.69^a^	450.84^a^	57.34	0.02
Chao	430.36^a^	277.60^b^	452.25^a^	472.63^a^	445.92^a^	59.08	0.03
Coverage	0.998	0.997	0.997	0.998	0.998	0.00	0.14

Shannon and Simpson indices are used to assess biodiversity of milk microbiota

Ace and Chao indices are used to assess the microbiotal richness

Coverage index are used to reflect species coverage

^abc^Means in the same row with significant difference (*P* < 0.05)

### Milk microbiota community composition at phylum, genus, species level

**Fig. 4:**
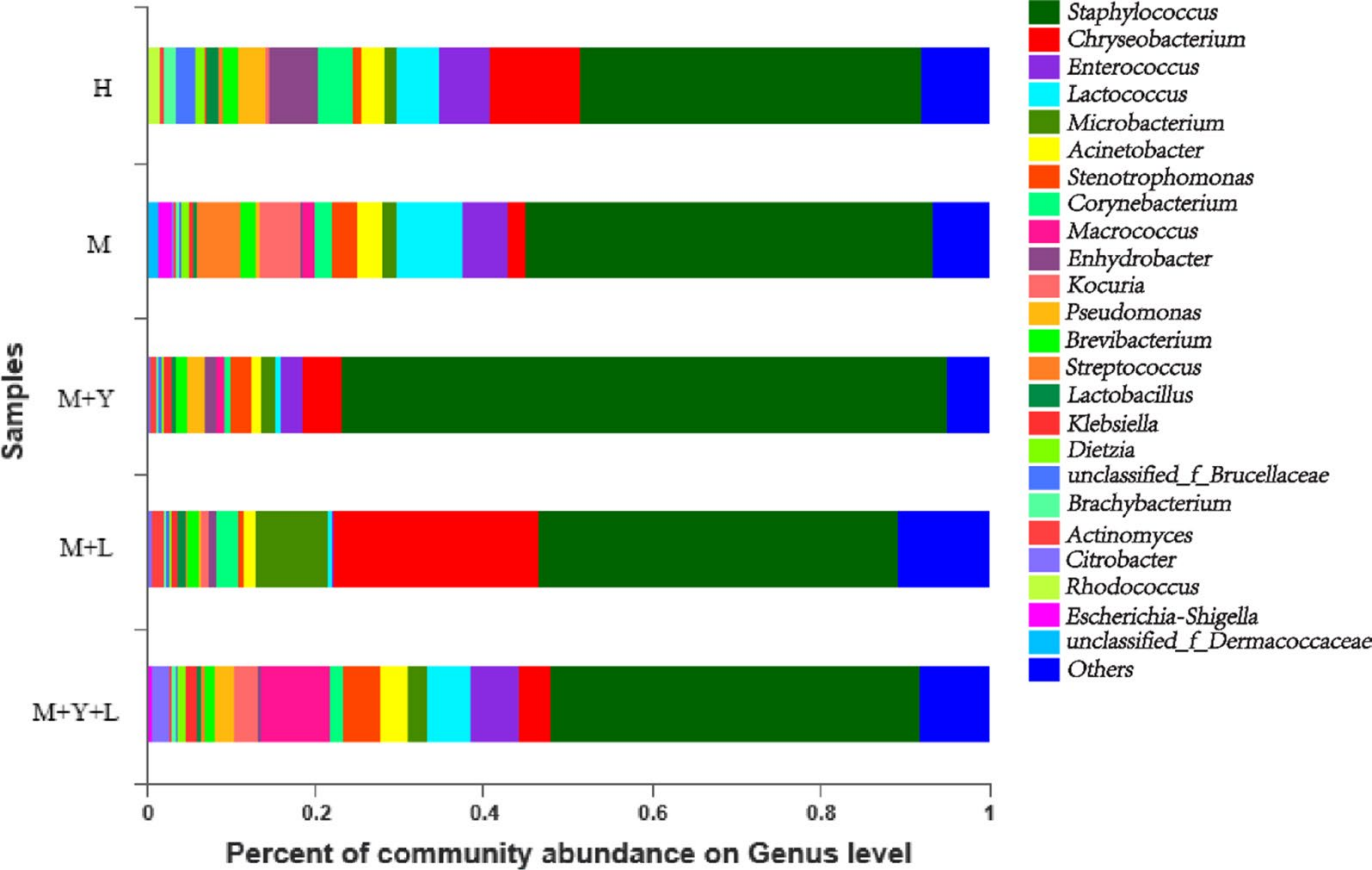
The effect of treatments on the distribution of milk bacterial community at the genus level

**Fig. 5:**
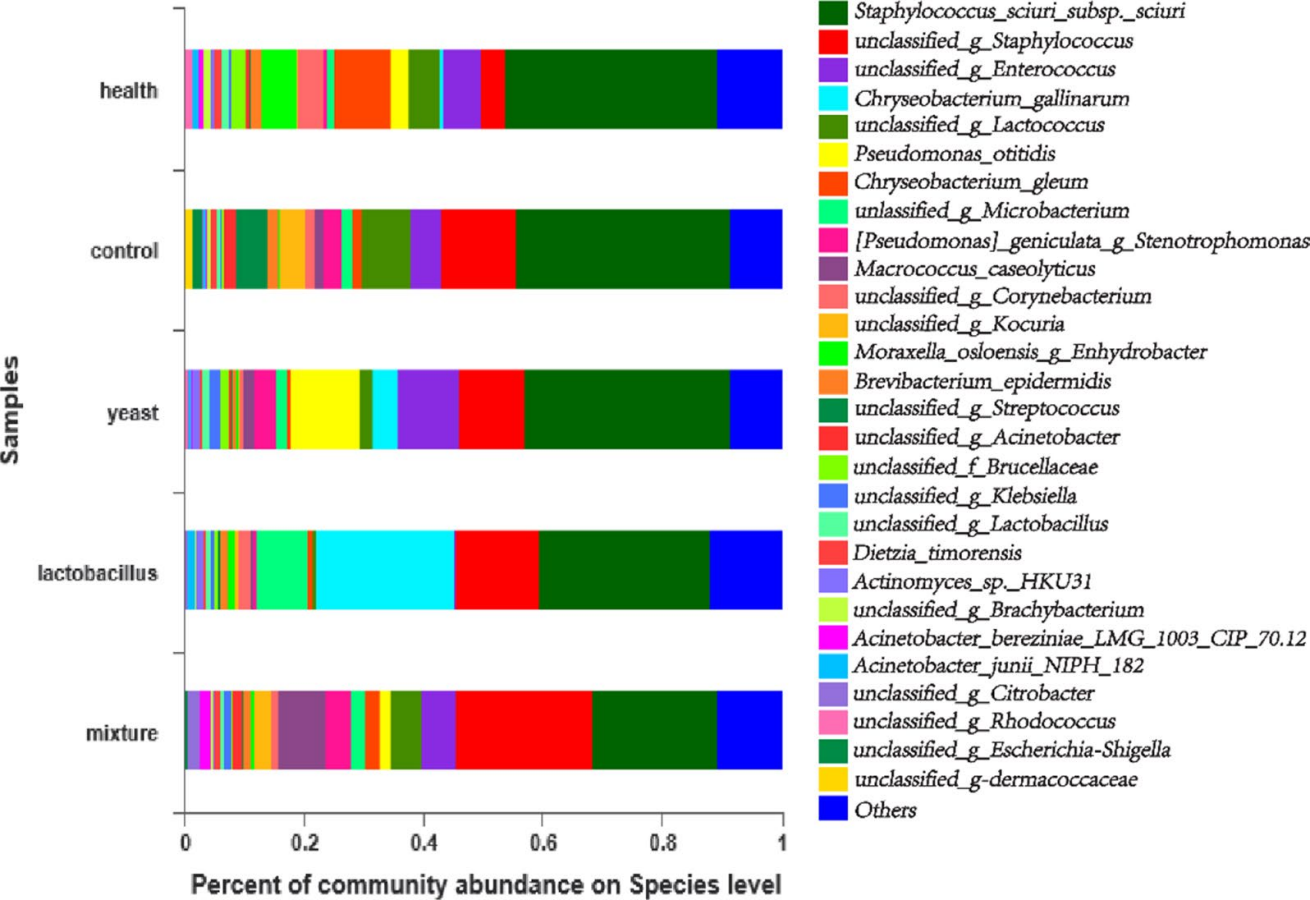
The effect of treatments on the distribution of milk bacterial community at the species level

*Firmicute* was the major phylum with a prevalence ranging from 72.46 to 42.54% (Additional file 1: Fig. S2), followed by *Proteobacteria* (17.67–10.45%), *Actionbacteria* (17.67–10.04%). As shown in Fig. 4, *Staphylococcus, Chryseobacterium* and *Lactococcus* were dominant genera among groups, but the abundance of genera was different. At species level, the effects of supplementation of LAB and yeast on milk microorganisms community abundance were shown in Fig. 5. The most prevalent microbial species were *Staphylococcus sciuri*, *Enterococcus* and *unclassified Enterococcus*, but the abundance were diverse among groups.

### Milk microbiota community composition difference between different groups

**Fig. 6:**
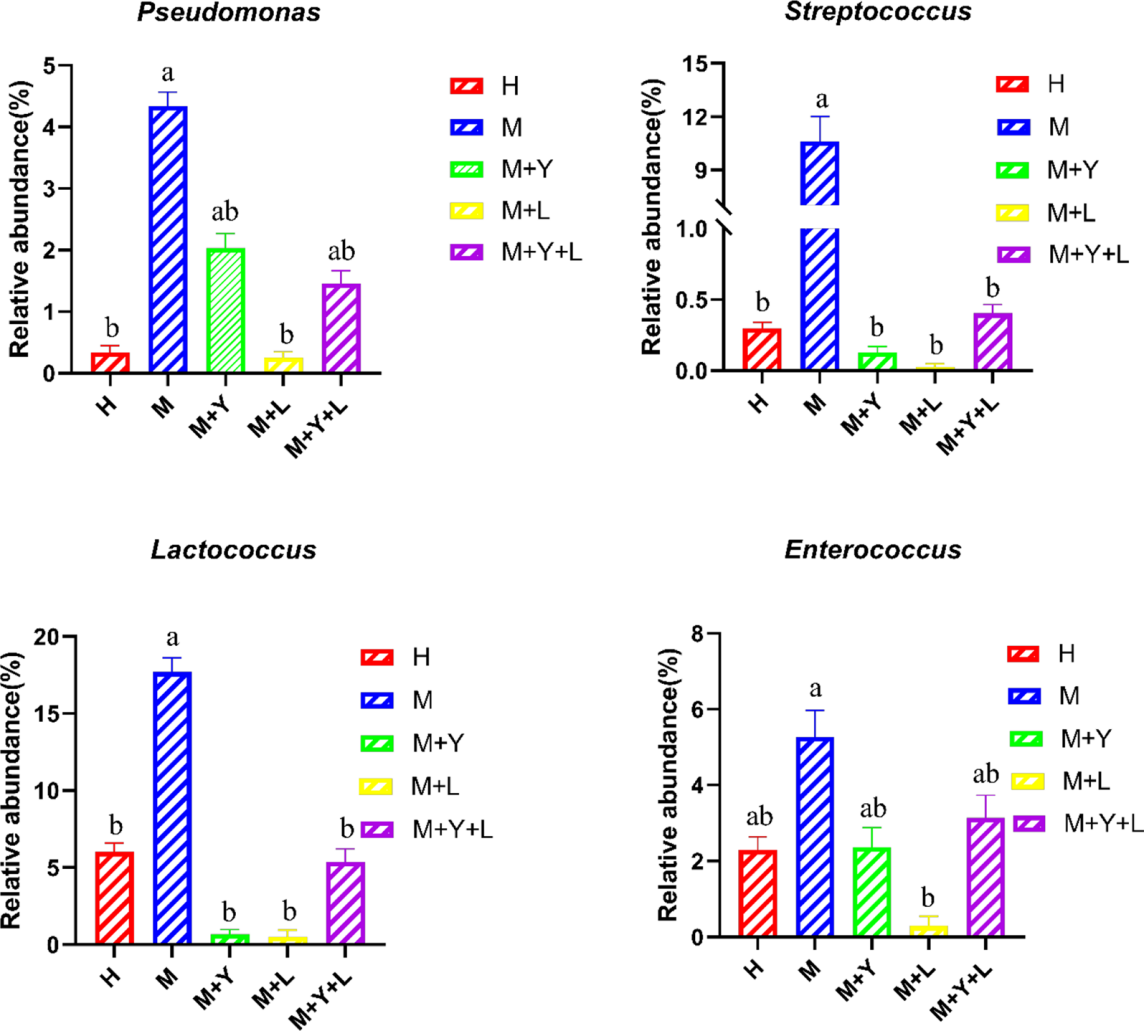
The relative abundance (%) of *Pseudomonas*, *Streptococcus*, *Lactococcus* and *Enterococcus* at genus level

We used one-way ANOVA and T test to identify the difference in microbiota composition between groups at genus level. As is shown in Fig. 6, treatments significantly changed the relative abundance of *Pseudomonas*, *Streptococcus*, *L.* and *Ent.* (*P *< 0.05). The relative abundance of *Ps.* in group M were significantly higher than that of group H, M + L (*P *< 0.05). Group M + Y and M + Y + L showed the middle value in relative abundance of *Ps.* (*P *> 0.05). Supplementation of probiotic decreased the relative abundance of *Strep.* compared with group M (*P *< 0.05), but there was no difference among group M + Y, M + L and M + Y + L (*P *> 0.05). The relative abundance of *L.* in group M were significantly higher than other groups (*P *< 0.05). The relative abundance of *Ent.* were highest in group M, lowest in group M + L (*P *< 0.05), and intermediate for H, M + Y, M + Y + L (*P *> 0.05).

### Microbial function prediction

**Fig. 7:**
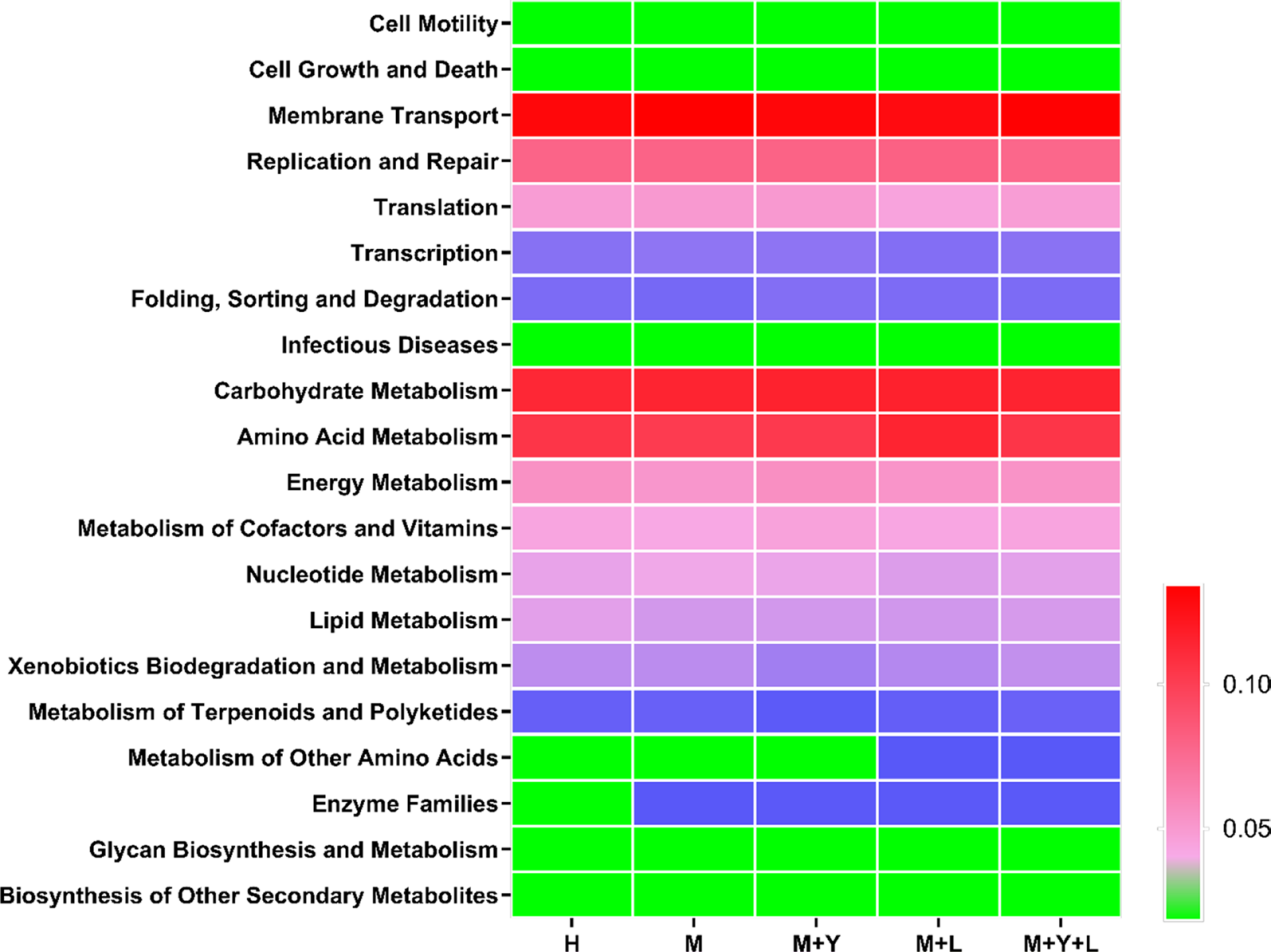
Top 20 abundant of Heatmap of KEGG from different treatments on level 2

To investigate the functional profiles of milk bacterial community, PICRUSt was used to analyze the KEGG pathway compositions in milk microbiota populations. A total of 39 level 2 KEGG pathway were detected in milk samples. The top 20 abundant level 2 KEGG pathway in milk microbiota were shown in Fig. 7. The most prevalent pathway involved in milk microbiota were membrane transport, followed by carbohydrate metabolism and amino acid metabolism. As shown in Table 4, the relative abundance of 13 pathway were significantly influenced by treatments (*P *< 0.05). Basically, relative abundance of immune system disease and metabolic disease pathway in group H, M + Y, M + L, M + Y + L were lower than that of group M (*P *< 0.05). Supplementation of LAB significantly decreased the relative abundance of infectious disease pathway and increased the relative abundance of immune system pathway (*P *< 0.05).

**Table 4:** The relative abundance of level 2 KEGG pathway of milk microbiota from different treatments

Items	Treatments					SEM	*P*-value
	H	M	M + Y	M + L	M + Y + L		
Human disease							
Cancers	0.1133^b^	0.0850^b^	0.1012^b^	0.1456^a^	0.1052^b^	0.0149	0.024
Immune system diseases	0.0519^b^	0.0629^a^	0.0475^b^	0.0541^b^	0.0529^b^	0.0034	0.012
Infectious diseases	0.4914^a^	0.5046^a^	0.5477^a^	0.3779^b^	0.4942^a^	0.0542	0.092
Metabolic diseases	0.0812^b^	0.0956^a^	0.0824^b^	0.0874^b^	0.0892^b^	0.0039	0.028
Neurodegenerative diseases	0.2609^a^	0.1935^b^	0.2158^b^	0.2304^a^	0.2048^b^	0.0193	0.049
Organismal systems							
Digestive system	0.0567^b^	0.0400^c^	0.0470^bc^	0.0767^a^	0.0391^c^	0.0069	0.002
Environmental adaptation	0.1018^b^	0.0960^b^	0.0992^b^	0.1194^a^	0.1028^b^	0.0052	0.010
Immune system	0.0361^b^	0.0363^b^	0.0259^b^	0.0628^a^	0.0341^b^	0.0091	0.022
Metabolism							
Biosynthesis of other secondary metabolites	0.7393^b^	0.7458^b^	0.6535^b^	1.0859^a^	0.7434^b^	0.1151	0.029
Enzyme families	1.8068^b^	1.8567^a^	1.8796^a^	1.8767^a^	1.8676^a^	0.0229	0.055
Environmental information processing							
Signal transduction	1.7126^b^	1.7509^b^	1.8880^a^	1.6072^c^	1.8691^a^	0.0503	0.001
Cellular process							
Signaling molecules and Interaction	0.1751^bc^	0.1972^ab^	0.1576^c^	0.2010^a^	0.1682^c^	0.0130	0.031
Transport and catabolism	0.3127^b^	0.2578^b^	0.2635^b^	0.3818^a^	0.2611^b^	0.0319	0.013

13 level 2 KEGG pathway of milk microbiota are influenced by treatments, which are classified into 5 classfications according to the pathway involved in

^abc^Means in the same row with significant difference (*P* < 0.05)

## Discussion

It is recently known that milk has its own microbiota and the mechanism probiotics exert benefit on milk microorganisms just starts. It is not clear that if oral intake of probiotics will pass from gut tract to mammary gland through endogenous route or pointing towards a systemic effect such as enhancing immune function, or both. Mastromarino et al. (2015) reported that oral administration of a multi-strain probiotic product (VSL#3) to women, consisting of *lactobacilli*, *bifidobacteri* and *Streptococcus thermophilus*, positively influenced milk microbiota through increased milk *lactobacilli* and *bifidobacteri* levels during late pregnancy and early lactation. Since high SCC and milk of inferior are the common indicator and consequence of mastitis for dairy cows, decreased SCC and improved milk quality in our study suggested the amelioration of mastitis. Higher milk percentage of fat and lactose caused by dietary supplementation of yeast has been reported in dairy goats (Ma et al. 2019) and dairy cows (Olagaray et al. 2019), while similar study with LAB are rare. Catozzi et al. (2019) reported that intramammary incubation of inactivated *Lactobacillus rhamnosus* caused significant increase of SCC on cows with subclinical at the beginning but decreased afterwards, as well as modulation of milk microbiota. Combined with our study, oral intake of LAB also decreased milk SCC and improved the milk quality in mastitis cows, indicating the relieving effects of LAB on mastitis.

The benefits probiotics exerted on mammary gland may be induced by relieving inflammation response. Supplementation of the mixture of LAB and yeast decreased the milk concentration of TNF-α, IL-1β and IL-6 compared with mastitis cows, while single use of yeast or LAB was unable to reduce the concentration of those inflammation cytokine concentration. Yuan et al. (2015) reported that 30 g day^−1^ and 60 g day^−1^ live yeast decreased uterine IL-6 mRNA of early lactation cows, which indicating modulated uterine inflammation signal. Similarly, study showed that adding LAB prevented *E. coli* infection and depressed the expression of proinflammation cytokine IL-8 and IL-1β of endometrial cells in vitro (Genis et al. 2016). MPO activity is a quantitative assessment of neutrophil infiltration into mammary gland (Kan et al. 2019), and our result showed that milk activity of MPO was significantly decreased by supplementation of yeast, LAB or the mixture. In general, combination of yeast and LAB had potential at regulating inflammation of bovine mammary gland.

During mastitis, mammary gland goes through immune response, which increases the permeability of tight junctions and compromises the milk–blood barrier (Wellnitz and Bruckmaier 2012). This causes the promoted passage of several blood components to milk. The increase appearance of LDH, ALP and NAG in milk are considered as indictors of mastitis (Chagunda et al. 2006; Guha et al. 2012). As expected, the milk activity of LDH, ALP and NAG of mastitis cows were higher than health cows, which indicates the destruction of blood–milk barrier. Compared with single supplementation of yeast or LAB, cows fed combination of yeast and LAB had the lowest milk NAG activity, suggesting the best repairing effects on milk–blood barrier. Once milk–blood barrier is damaged, gram-native pathogens enters the systemic calculation and triggers immune response, which illustrates the increased in IgA and IgM in our results (Wall et al. 2016). IgG is the major immunoglobulin contained in milk, which transfer more through blood–milk barrier because of increased permeability. According to the results, Supplementation of single yeast or the mixture of yeast and LAB decreased the serum level of IgA, IgG and IgM, but the value was still higher than health cows, suggesting improved immune condition. Our results were not consistent with Geng et al. (2018), who reported that supplementation of yeast didn’t affect serum contents of IgA, IgM and IgG.

In the present study, we observed that the common bacteria OTUs shared by five groups were 282, which accounted 31.82% of the total and suggested the dynamics of milk microbiota communities among five groups. Beta diversity analysis showed different composition of the milk microbiota among caused by treatments. The most abundant phylum in our trial is *Firmicutes* and *Proteobacteria*, which is in accordance with Patel et al. (2016). At genus level, our results are in partial agreement with observation of Derakhshani et al. (2018), who reported that *Staph.* was the most abundant bacterial genus throughout the first week of lactation. *Staph.* are predominant colonizer of bovine mammary gland commonly considered to be major pathogen responsible for subclinical mastitis (Condas et al. 2017). *Chryseobacterium*, another dominant Gram-negative bacteria, are known for its ability to facilitate the spoilage pasteurized milk and recontamination, which lowers the milk quality (Schmidt et al. 2012). *Ent.,* followed by *L*, are reported as opportunistic pathogen causing mastitis in dairy cows (Oikonomou et al. 2014).

At genus level, supplementation of LAB lowered the mean proportions of *Ps.*, which is the main strain responsible for the spoilage of refrigerated raw milk. Many species of *Ps.* are able to produce extracellular peptidases and lipases that remain active during thermal process and damage technological performances of milk and deterioration of the dairy products (Decimo et al. 2014). Similar to our results, D’Amico de Alcantara et al. (2019) also reported *Lactobacillus rhamnosus* inhibited the growth of *Ps.* in inoculated milk samples. LAB exert antimicrobial activity against the growth of deteriorating and pathogenic microorganisms through the production of various antimicrobial compounds, including bacteriocins, organic acids, and hydrogen peroxide (Prabhurajeshwar and Chandrakanth 2017; Todorov and Dicks 2005). According to our results, supplementation of mixture of yeast and LAB didn’t reduce the mean proportions of *Ps.*, which may result from the different dose of LAB. *Strept.* is one of the major pathogens causing mastitis in dairy cows, mainly *Streptococcus agalactiae*, *Streptococcus dysgalactiae*, and *Streptococcus uberis* (Coffey et al. 2006). Our results showed the decreased mean proportion of *Strep.* after supplementation of probiotic, which indicates the relived mastitis. In agreement with our data, Nasiri et al. (2019) reported that supplementation of 4 g day^−1^ of live yeast fostered the lymphocyte proliferative response in transition dairy cows, which indicates an improved immune function. Thus, antimicrobial effects of yeast on mastitis cows may be associated with enhanced systemic immune function. *L.*, which is considered a commensal species, has also been detected in raw milk sampled from healthy bovine mammary glands (Lafarge et al. 2004). In our trial, the mean proportions of *L.* in group M was higher than that of other groups, which is consistent with Patel et al. (2019) who reported that milk samples from subclinical mastitis cow had higher abundance of *L.* compared to healthy cows. Some strains of *L.* exhibit high adherence to bovine mammary epithelial cells, which means *L.* can also enter mammary gland of mastitis cows through teat and contaminant with pathogenic bacterial (Hagi et al. 2013). *Ent.*, especially *Enterococcus faecium* and *Enterococcus Faecalis*, is the predominant environmental mastitis causing pathogen (Gao et al. 2019). *Ent.* are commonly found in animal intestines and their feces and characterized by a high level of resistance to many antibacterial substances, which limites the therapeutic result of antimicrobials (Rozanska et al. 2019). In our trial, supplementation of LAB exhibited inhibitory effect on abundance of *Ent.*, indicating antimicrobial activity against mastitis. Based on KEGG pathway analysis of milk samples, supplementation of LAB also decreased relative abundance of pathway in immune system disease and infectious disease, while increased relative abundance of pathway in immune system, all of which are associated with mastitis and inflammation.

Supplementation of LAB and yeast show potential of relieving, while supplementation single strain LAB had best relieving the mammary inflammation and changing the milk microorganisms.

## Supplementary information

**Additional file 1: Fig. S1.** Shannon index of OTU level of milk microbiota on different treatments. **Fig. S2.** The effects of treatments on the distribution of milk bacterial community at the phylum level

## Data Availability

All sequences analyzed in this study can be accessed in Zenodo database (http://doi.org/10.5281/zenodo.3384327). The data supporting the conclusion of this article are included in this article.
