# Supplementary material for: Impact of yeast and lactic acid bacteria on mastitis and milk microbiota composition of dairy cows
Source: AMB Express. 2020 Jan 29;10:22. doi: 10.1186/s13568-020-0953-8 (PMC6987887; doi:10.1186/s13568-020-0953-8)
Supplement: Supplementary file 1 — Additional file 1: Fig. S1. Shannon index of OTU level of milk microbiota on different treatments. Fig. S2. The effects of treatments on the distribution of milk bacterial community at the phylum level [file 13568_2020_953_MOESM1_ESM.docx]

**AMB Express**

**Impact of yeast and Lactic Acid Bacteria on mastitis and milk microbiota composition on dairy cows**

**Jing Gao^1^, Yu-Chen Liu^1^, Yu Wang^1^, Han Li^1^, Xiang-Ming Wang^1^, Yan Wu^1^, Ding-Ran Zhang^1^, Si Gao^1^, Zhi-li Qi^*1^**

^1^Department of Animal Nutrition and Feed Science, College of Animal Science and Technology, Huazhong Agricultural University, Wuhan, China

**^*^**Corresponding author: Zhi-li Qi, Associate Professor, Department of Animal Nutrition and Feed Science, College of Animal Sciences and Technology, Huazhong Agricultural University, Wuhan, China, Tel: +86 13296621998, Email: [zhiliqi@mail.hzau.edu.com](mailto:zhiliqi@mail.hzau.edu.com)

Fig. S1 Shannon index of OTU level of milk microbiota on different treatments

Fig. S2 The effects of treatments on the distribution of milk bacterial community at the phylum level


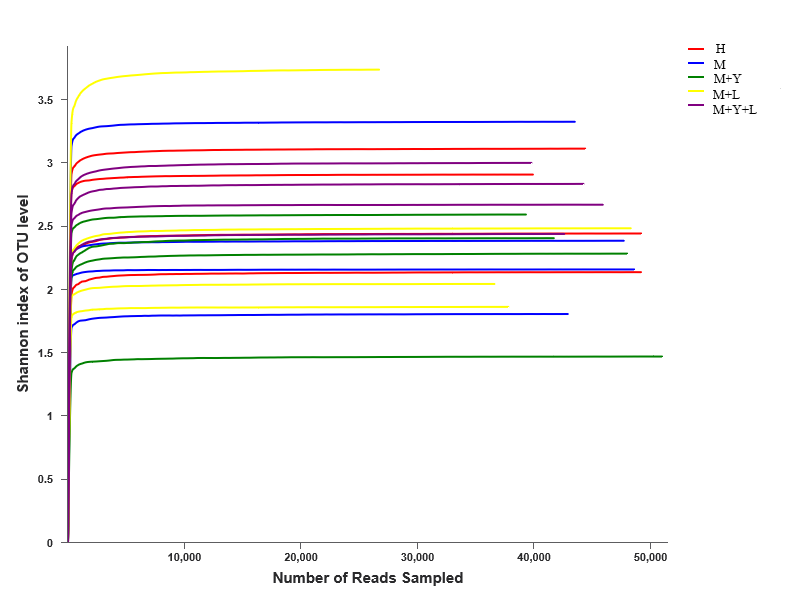


Fig. S1


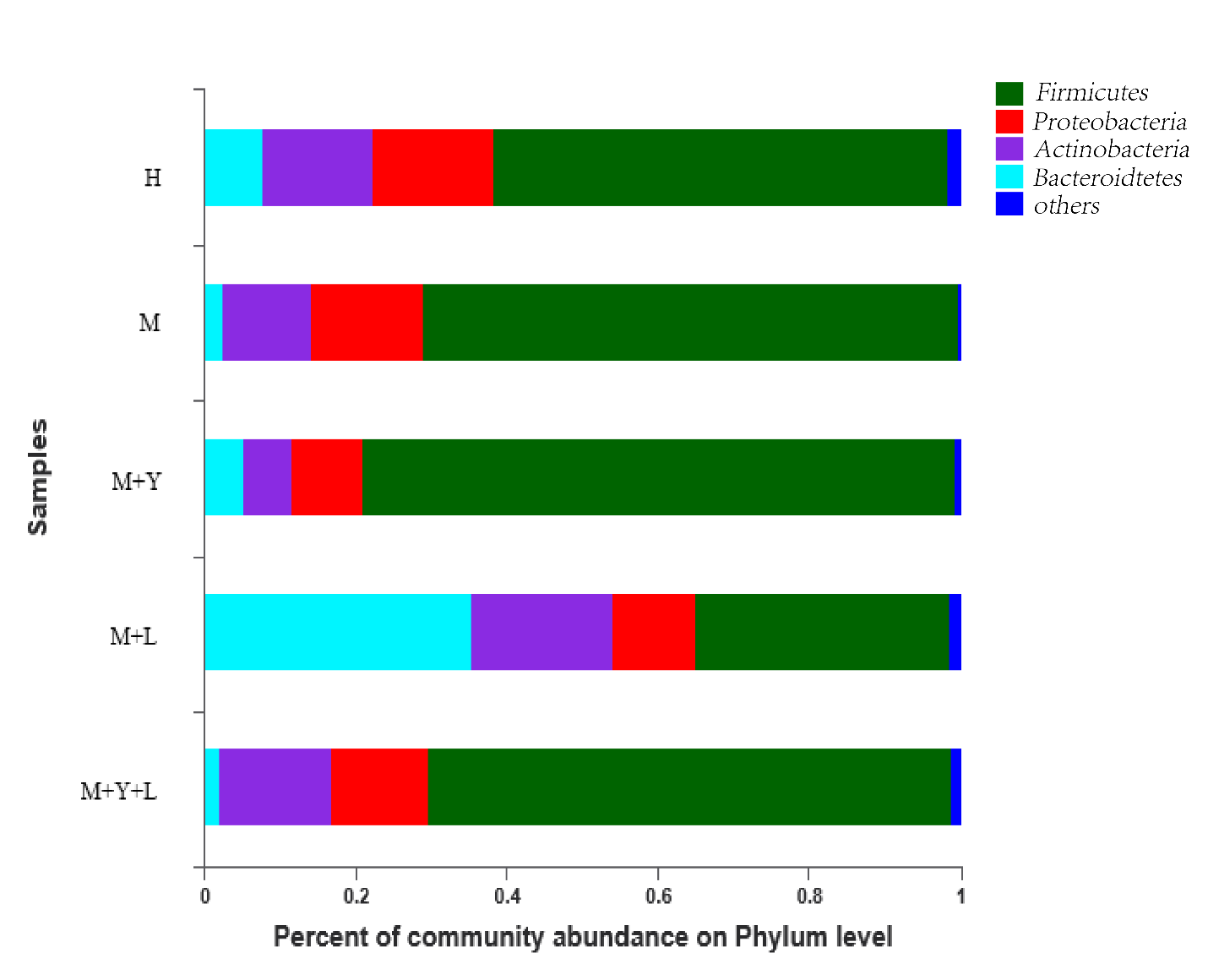


Fig. S2
